# Therapeutic efficacy of sulforaphane in autism spectrum disorders and its association with gut microbiota: animal model and human longitudinal studies

**DOI:** 10.3389/fnut.2023.1294057

**Published:** 2024-01-08

**Authors:** Jiexian Yang, Li He, Si Dai, Huihui Zheng, Xilong Cui, Jianjun Ou, Xiaojie Zhang

**Affiliations:** Department of Psychiatry, National Clinical Research Center for Mental Disorders, and National Center for Mental Disorders, The Second Xiangya Hospital of Central South University, Changsha, Hunan, China

**Keywords:** sulforaphane, autism spectrum disorders, gut microbiota, animal model, clinical study

## Abstract

**Introduction:**

Sulforaphane (SFN) has been found to alleviate complications linked with several diseases by regulating gut microbiota (GM), while the effect of GM on SFN for autism spectrum disorders (ASD) has not been studied. Therefore, this study aimed to investigate the relationship between the effects of SFN on childhood ASD and GM through animal model and human studies.

**Methods:**

We evaluated the therapeutic effects of SFN on maternal immune activation (MIA) induced ASD-like rat model and pediatric autism patients using three-chamber social test and OSU Autism Rating Scale-DSM-IV (OARS-4), respectively, with parallel GM analysis using 16SrRNA sequencing.

**Results:**

SFN significantly improved the sniffing times of ASD-like rats in the three-chamber test. For human participants, the average verbal or non-verbal communication (OSU-CO) scores of SFN group had changed significantly at the 12-wk endpoint. SFN was safe and no serious side effects after taking. GM changes were similar for both ASD-like rats and ASD patients, such as consistent changes in order *Bacillales*, family *Staphylococcaceae* and genus *Staphylococcus*. Although the gut microbiota composition was significantly altered in SFN-treated ASD-like rats, the alteration of GM was not evident in ASD patients after 12 weeks of SFN treatment. However, in the network analysis, we found 25 taxa correlated with rats' social behavior, 8 of which were associated with SFN treatment in ASD-like rats, For ASD patients, we found 35 GM abundance alterations correlated with improvements in ASD symptoms after SFN treatment. Moreover, family *Pasteurellaceae* and genus *Haemophilus* were found to be associated with SFN administration in the network analyses in both ASD-like rats and ASD patients.

**Discussion:**

These findings suggest that SFN could provide a novel avenue for preventing and treating ASD, and its therapeutic effects might be related to gut microbiota.

## 1 Introduction

Autism spectrum disorder (ASD) is a neurodevelopmental disorder characterized by verbal or non-verbal expression difficulties, social difficulties, abnormal narrow interests, and continuous repetitive movements. According to the Autism and Developmental Disabilities Monitoring Network, the prevalence of ASD was 2.3% among children aged 8 years in the US, with boys affected more frequently than girls, 3.7% (95% CI, 3.5–3.8) in boys and 0.9% (95% CI, 0.8–0.9) in girls (1). Currently, no medications have demonstrated efficacy for the core diagnostic symptoms of ASD (2), and behavioral interventions remain the primary means of treatment. Therefore, there is an urgent need to develop and research therapies that target core symptoms.

SFN, derived from broccoli, has gained attention for its health benefits, including its potential in treating cancer and cardiovascular disease (3). Recently, clinical studies have shown improvements in ASD patients treated with SFN. In a double-blind randomized trial with young men (aged 13–27) with ASD, SFN treatment led to significant behavioral improvements, with a 34% decline in Aberrant Behavior Checklist scores and a 17% decline in Social Responsiveness Scale scores (4). Similar positive effects were observed in children and young adults (aged 3–12) using a broccoli seed extract (5) and a randomized clinical trial (*N* = 108) in China (6). However, a study of children aged 3–7 with ASD showed inconsistent results, with no significant clinical improvement (7). Basic research studies about the mechanism of SFN in treating ASD were focused on redox metabolism (8), oxidative stress (9), mitochondrial dysfunction (10), immune dysregulation, neuroinflammation (11, 12), febrile illness (13), heat shock response (14), and synaptic dysfunction (15, 16). However, the exact therapeutic mechanism is still unclear.

Evidence suggested a bilateral influence between ASD and the gut microbiome (17). Individuals with ASD exhibit distinct gut bacterial communities, with a higher abundance of *Bacteroides, Parabacteroides, Clostridium, Faecalibacterium*, and *Phascolarctobacterium* and a lower abundance of *Coprococcus* and *Bifidobacterium*. (18). Some of them (*Bacteroides, Parabacteroides, Coprococcus*, and *Bifidobacterium*) have consistent changes in rat models of ASD (19–22). Furthermore, a recent study showed that autism-like behavior can be transferred to germ-free mice by transplanting fecal microbes from children with ASD (23). Additionally, interventions targeting the gut microbiome have shown promise in alleviating ASD symptoms. For example, oral vancomycin resulted in short-term benefits in a small group of children with ASD (24), and microbiota transfer therapy (MTT) altered the gut microbiome and improved GI and behavioral symptoms in children with ASD (25). Rat models of ASD have also demonstrated that *Bacteroides fragilis* (26) and *Lactobacillus reuteri* (27) could modulate gut microbiota and improve ASD-associated behaviors. All of this evidence suggests that gut microbes might play an important role in modulating brain function and behavior of ASD. Moreover, SFN has been shown to alleviate complications linked with several diseases in animal studies by modulating the gut microbiome. For example, SFN treatment increased the abundance of gut microbiome, such as *Butyricicoccus* in a mouse model of ulcerative colitis (28, 29), normalized dysbacteriosis bacteria in a bladder cancer mice model (30), and altered the relative abundance of disease-associated microbial species in a hyperuricemia rat model, particularly by increasing the abundance of *Saccharomyces*, Lactobacillaceae, and Clostridiaceae and decreasing the abundances of *Bacteroides, Parasutterella*, and *Alistipes* (31). SFN also reduced body weight, liver inflammation, and hepatic steatosis in high-fat diet mice by modulating the gut microbiota (32). These studies indicated the impact of SFN on the gut microbiome, but no studies have yet explored whether SFN can alleviate ASD symptoms by regulating the gut microbiome.

To investigate the therapeutic effect of SFN treatment on childhood ASD and its potential relationship with GM, we constructed a younger age ASD animal model using MIA-induced ASD-like rat model, evaluated the therapeutic effect of SFN, and investigated the potential role of GM by three-chamber social test and 16S rRNA sequencing, respectively. Meanwhile, we also conducted a longitudinal study to investigate the therapeutic effects of SFN on childhood ASD by recruiting patients aged 4–7 years with ASD and to explore the potential relationship between the therapeutic effects of SFN and GM. In summary, this study aims to reveal the role of SFN in the treatment of ASD in children and its potential relationship with GM through both clinical and animal studies and to provide new insights into the treatment and pathogenesis of ASD.

## 2 Materials and methods

### 2.1 Animal and ASD-like rat model

Sprague–Dawley rats were purchased from Hunan Silaike Jingda Laboratory Animal, China. Animals were housed in groups of five per cage under a regular 12-h light/dark cycle and with access to food and water *ad libitum*. The rats were fed by the same type of food and lived in the same environment. The maternal immune activation (MIA)-induced ASD-like rat model was employed as previously described (33, 34). Adult pregnant female Sprague-Dawley rats were randomly divided into two groups and were intraperitoneal injected with lipopolysaccharide (LPS; 200 μg/kg) in saline or saline vehicle (veh, 0.9% NaCl) on gestational days 12 and 15, respectively. On postnatal day 25, the newborn male rats were separated and on day 40 were subjected to the three-chamber test to confirm autistic-like features (LPS modeled group: *n* = 38; saline group: *n* = 10). Then, the LPS modeled group and the saline group were divided into two subgroups, which were intraperitoneal injected with SFN 20 ug/kg or vehicle control, respectively, for another 28 days: LPS modeled group treated by SFN (LPS-SFN): *n* = 19; LPS modeled group treated by saline (LPS-NS): *n* = 19; saline group treated by SFN (NS-SFN): *n* = 5; saline group treated by saline (NS-NS): *n* = 5. After the treatment, all four groups of rats were subjected to the three-chamber test. The Animal Users Care and Use Committee of Central South University approved our experimental protocol, and the ethics approval number is 20200006.

### 2.2 Three-chamber test

**Figure 1:**
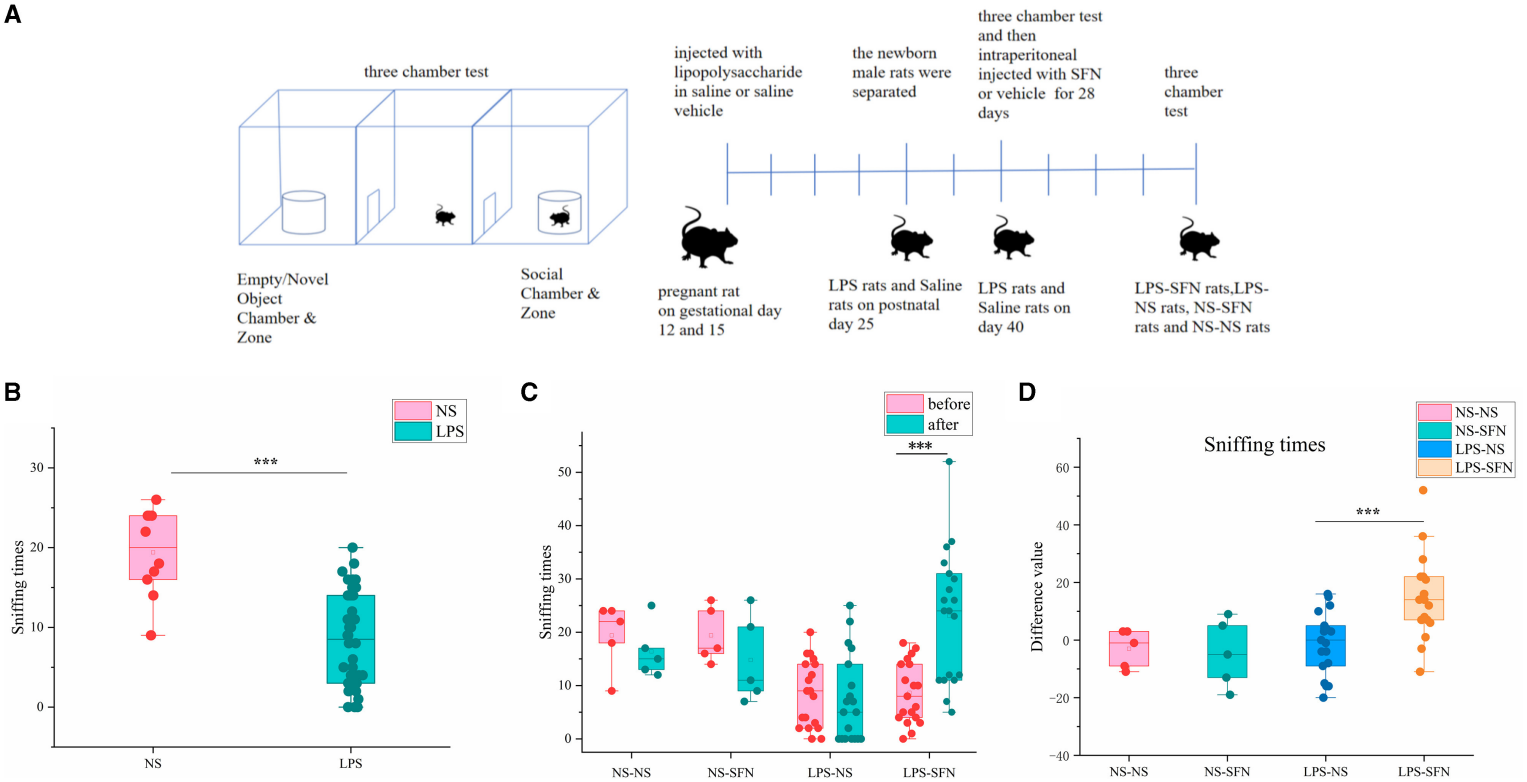
Behavioral assessment of MIA-induced ASD-like rat model before and after SFN treatment. **(A)** Flow diagram of experiment. **(B)** Sniffing time of MIA-induced ASD-like rats (LPS) vs. control rats (NS) in the three-chamber test before SFN or saline treatment. **(C)** The sniffing times of different groups of rats in the three-chamber test (before or after the administration of saline or SFN). **(D)** Difference value in sniffing times after-before the administration of saline or SFN (***p < 0.001, paired Students' *t*-test or unpaired Students' *t*-test). Difference value: rats' sniffing times after the administration of saline or SFN were subtracted from the same rat's sniffing times before the administration.

The three-chamber apparatus used in the experiment consisted of a non-transparent plexiglass box with two transparent partitions creating left, center, and right chambers (40 × 60 cm). Each partition had a square opening (10 × 10 cm) in the bottom center. A wire cage (18 cm diameter) was used as an inanimate object or to house a stranger rat, with a water-filled bottle placed on top to prevent the test rat from climbing. The unit and wire cups were cleaned with 70% ethanol between each trial. In the first 10-min session, the test rat explored the empty chambers. In the second 10-min session, a stranger rat was placed in one of the wire cages, and in the last 10-min session, a second stranger rat was placed in the other wire cage (Figure 1A). The movement of the rat was recorded by a USB webcam (LifeCam HD-6000, Microsoft) and PC-based video capture software (WinAVI Video Capture, ZJ Media Digital Technology). The recorded video file was further analyzed by offline video tracking software (EthoVision XT 7.0). The sniffing times to each wire cage were measured. We performed two three-chamber tests on rats, the first at 40 days postnatal and the second at 68 days postnatal, when rats were administered SFN or saline for 28 consecutive days.

### 2.3 Clinical trial design

The data and stool samples of ASD patients were obtained from previous clinical trials (6), and healthy controls were recruited by age-matched. Eleven healthy controls and six individuals with ASD were recruited by the Department of Psychiatry, Second Xiangya Hospital, which is affiliated with Central South University in Changsha, Hunan Province of China. There were no significant differences in age between the healthy control and ASD patients. All the recruited subjects were boys. In the present study, we included 4- to 7-year-old children with ASD diagnosed based on the following criteria: (1) 4- to 7-year-old only; (2) met Diagnostic and Statistical Manual of Mental Disorders, Fifth Edition (DSM-V) diagnostic criteria for ASD; (3) re-confirmed using Autism Diagnostic Interview-Revised (ADI-R) and Autism Diagnostic Observation Schedule (ADOS). The exclusion criteria were as follows: (1) severe physical diseases (i.e., thyroid disease and congenital heart disease); (2) known history of ASD-associating genetic syndromes (i.e., Fragile-X syndrome and Down's syndrome); (3) severe brain diseases (i.e., epilepsy and brain trauma); (4) absence of neurodevelopmental disorders and other psychiatric disorders as assessed by a child psychiatrist.

After screening, participants with ASD were treated by SFN for 12 weeks. We obtained fecal samples of both the healthy control group and the ASD group at baseline (ASD-Baseline) and the ASD group on the last day of week 12 (ASD-SFN). Guardians of all participants received information about the protocol and provided an informed consent form before enrollment. The study was approved by the ethics committee of Second Xiangya Hospital and was registered at ClinicalTrials.gov (NCT02879110).

### 2.4 Medication intervention, safety measures, and behavioral outcome measures

SFN was delivered as Avmacol^®^ (Nutramax Laboratories, Inc., Edgewood, Maryland, USA) tablets which contain both glucoraphanin and active myrosinase enzyme and are formulated to support sulforaphane production from ≥30 μmol of glucoraphanin per tablet. Tablets were maintained at room temperature and checked periodically microbiologically. The dose of SFN was based on weight. SFN was safe and no serious side effects after taking. Changes in the OSU Autism Rating Scale-DSM-IV (OARS-4) were the priority primary outcome measurement. OARS-4 consists of three domains, namely, social interaction (OSU-SO), verbal or non-verbal communication (OSU-CO), and repetitive or ritualistic behaviors (OSU-ST), and their average scores stand for the total behavioral outcome (OSU-total). The score of the ASD group was assessed at baseline and on the last day of week 12. Refer to our previous clinical studies for specific SFN usage, safety measures, and OARS-4 measures (6).

### 2.5 Fecal sample collection

Fecal samples from rats were collected uniformly after the three-chamber test was done, while fecal samples from clinical participants were collected before (baseline) and after 12 weeks of SFN treatment. All fecal samples were collected into sterile tubes and rapidly frozen with liquid nitrogen. It is stored in a refrigerator at −80°C until use.

### 2.6 DNA extraction, PCR amplification, and 16S rRNA gene sequencing

DNA was extracted from stool samples using the E.Z.N.A^®^ DNA kit (Omega Bio-Tek, USA). The V3–V4 regions of the bacteria 16S rRNA gene were amplified by polymerase chain reaction (PCR) (98°C for 5 min, followed by 25 cycles consisting of denaturation at 98°C for 30 s, annealing at 53°C for 30 s, and extension at 72°C for 45 s, and a final extension at 72°C for 5 min). PCR amplicons were purified using Vazyme VAHTSTM DN A Clean Beads (Vazyme, N Nanjing, China China), quantified using the Quant-iT PicoGreen dsDNA Assay Kit (Invitrogen, Carlsbad, CA, USA), and sequenced using the Illumina MiSeq platform with MiSeq Reagent Kit v3 at Shanghai Personal Biotechnology Co., Ltd (Shanghai, China). Purified amplicons were pooled at equimolar concentrations and sequenced (2 × 300) on the MiSeq platform (San Diego, USA) according to the standard protocol of Mariobio Biomedical Technologies (Shanghai, China).

### 2.7 Processing of sequencing data

Quality filtering at QIIME (version 1.9.1) was followed by first demultiplexing, using the previously used set of validation criteria: (i) Quality fraction checks were performed with 300 bp reads at any site to obtain an average quality fraction <20 over a 50 bp sliding window; truncated reads <50 bp were discarded. (ii) Removal of exact barcode matches, two nucleotide mismatches in primer matches, and reads containing ambiguous characters. (iii) Sequence assembly, where only sequences with overlap lengths >10 bp are assembled based on the overlapping sequences. Reads that cannot be assembled will be discarded.

Operational taxonomic units (OTUs) were clustered using UPARSE (version 7.1) with a 97% cutoff threshold, 19 sequences were identified, and chimeric sequences were removed using UCHIME.20. For each 16S rRNA gene sequence, the SILVA (SSU123) 16S rRNA database was analyzed for classification using the RDP classifier21 with a 70% confidence threshold.

To improve downstream statistical analysis, we removed low-quality or uninformative features by low count filter and low variance filter. The cutoff threshold of low count filter was set at 10% prevalence filter which means at least 10% of its values should contain at least two counts and the low variance filter was measured by inter-quartile range (IQR).

### 2.8 Network analysis

Network analysis for the GM and sniffing times and SFN treatment or clinical ASD symptoms was performed using Spearman's rank correlations conducted by IBM SPSS Statistics 27.0.1 and network reconstruction and property measurements conducted by Gephi 0.9.7. We first computed the correlation between each node, and only statistically significant Spearman's rank correlations (*p* < 0.05) were defined as an edge of two nodes. We next constructed undirected network graphs to display the potential relationship between GM and social features in MIA-induced ASD-like rat models or ASD patients using Gephi 0.9.7.

### 2.9 Statistical analysis

Richness (ACE and Chao1) and diversity (Shannon and Simpson) were used to assess the α-diversity indexes. Principal coordinate analysis (PCoA) of weighted and unweighted UniFrac22 was used to visualize the clustering patterns between samples based on β-diversity distances via R language. ANOSIM test was performed to identify differences in β-diversity among groups. Identification of key gut microbiota responsible for the differentiation between taxa using the effective size of linear discriminant analysis (LDA), only LDA > 1.5 at a *p* < 0.05 were considered significantly enriched. Multiple group comparisons were performed using one-way analysis of variance (ANOVA) followed by LSD as a *post-hoc* test and two-tailed Student's *t*-test or Mann–Whitney *U*-test to determine the difference between the two groups. All correlations were calculated using Spearman's correlation. Statistical analyses were conducted using the software SPSS, R package, and plots were generated from R and GraphPad Prism version 8.0. A *p*-value of 0.05 is significant for the test above. All data were analyzed using two-way ANOVA with Bonferroni's *post-hoc* analysis and one-way ANOVA with Tukey's *post-hoc* analysis.

## 3 Results

### 3.1 SFN treatment rescued the social deficits of MIA-induced ASD-like rat model

We first employed the MIA-induced ASD-like rat model and validated it by a three-chamber test. In the third 10-min test of the three-chamber test, sniffing times for novel rats in the LPS group were significantly less than that in the NS group (Figure 1B, *p* < 0.001). The results showed that rats in the LPS group demonstrated social deficits, indicating the successful establishment of our MIA-induced ASD-like rat model. Then, the LPS group and the saline group were subgroup into two groups and treated by SFN or saline, respectively. SFN treatment markedly rescued the reduced-sniffing times (Figure 1C; *p* < 0.001) of the LPS modeled group toward the novel rats, suggesting the rescued impaired social ability of SFN treatment. Comparing the difference value in Sniffing times before and after saline administration in the LPS-NS group, the difference value in Sniffing times before and after the administration of SFN was higher in the LPS-SFN group. It suggested that SFN treatment significantly increased the Sniffing times in MIA-induced ASD-like rats (Figure 1D, *p* < 0.001). These results indicated that our LPS modeling was successful and that SFN could reverse the socially deficient behavior of LPS rats.

### 3.2 Diversity of gut microbiota significantly altered in ASD-like rat model

**Figure 2:**
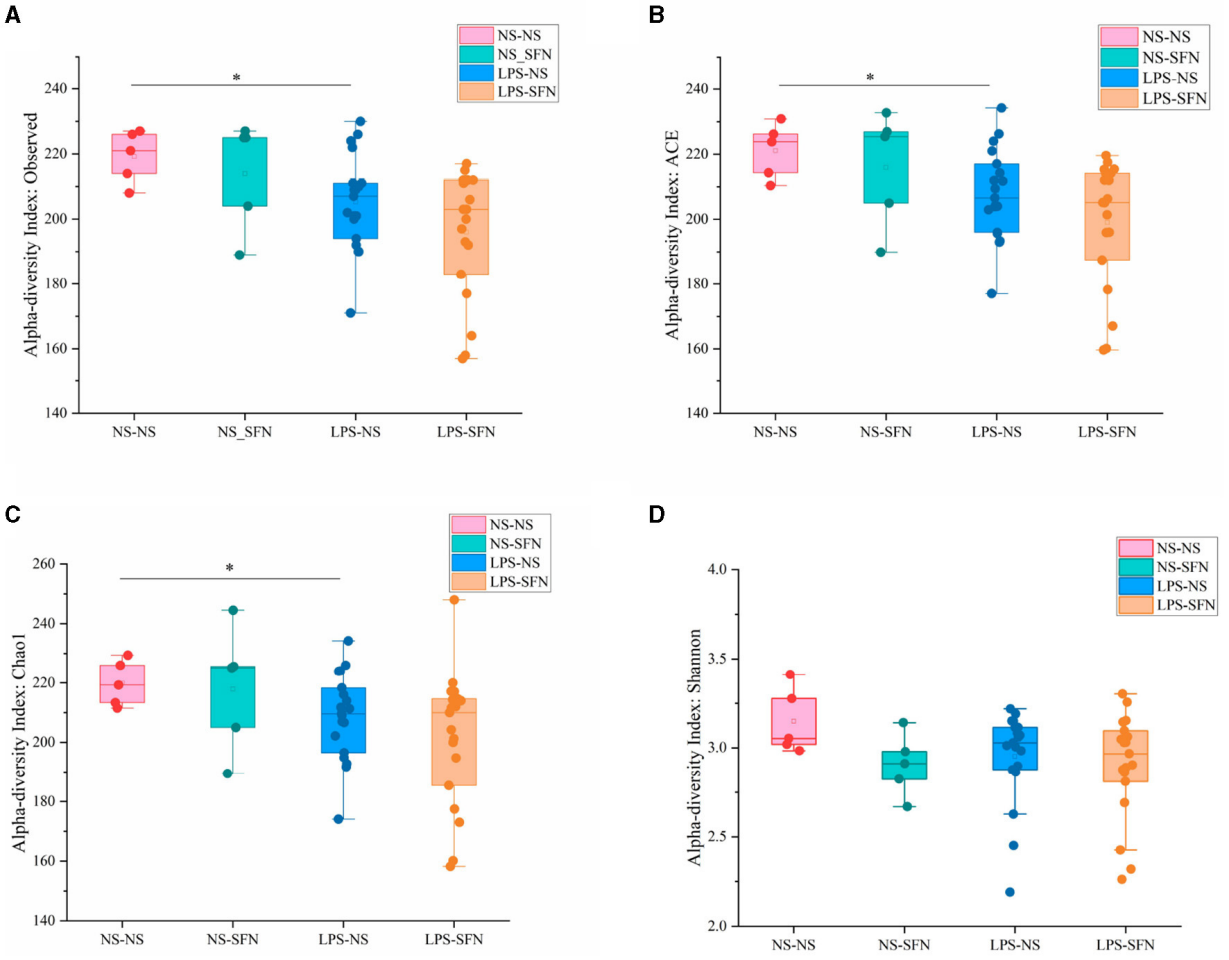
Diversity analysis of MIA-induced ASD-like rat model with or without SFN treatment. The observed operational taxonomic units (OTUs) **(A)**, ACE **(B)**, Chao1 **(C)**, and Shannon **(D)** used the Student's *t*-test (*p < 0.05).

We used 16S rRNA sequencing to evaluate alpha and beta diversity to determine differences in gut microbiota diversity. The alpha diversity was based on the numbers of observed OTUs, richness (ACE and Chao1), and diversity (Shannon and Simpson) (Figure 2). Observed OTU, ACE, and Chao1 indicated less microbiota alpha diversity in the LPS-NS group compared to the NS-NS group (*p* = 0.02, *p* = 0.03, and *p* = 0.03, respectively, Figure 2), but SFN treatment did not increase in alpha diversity in LPS rats. To evaluate the β-diversity of gut microbiota across different groups, principal coordinate analysis (PCoA) based on the unweighted UniFrac distance matrixes was conducted. Beta diversity assessed by the ANOSIM tests found the four groups could not cluster into distinct groups.

### 3.3 Changes of gut microbiota taxonomic composition in ASD-like rats and after SFN treatment

**Figure 3:**
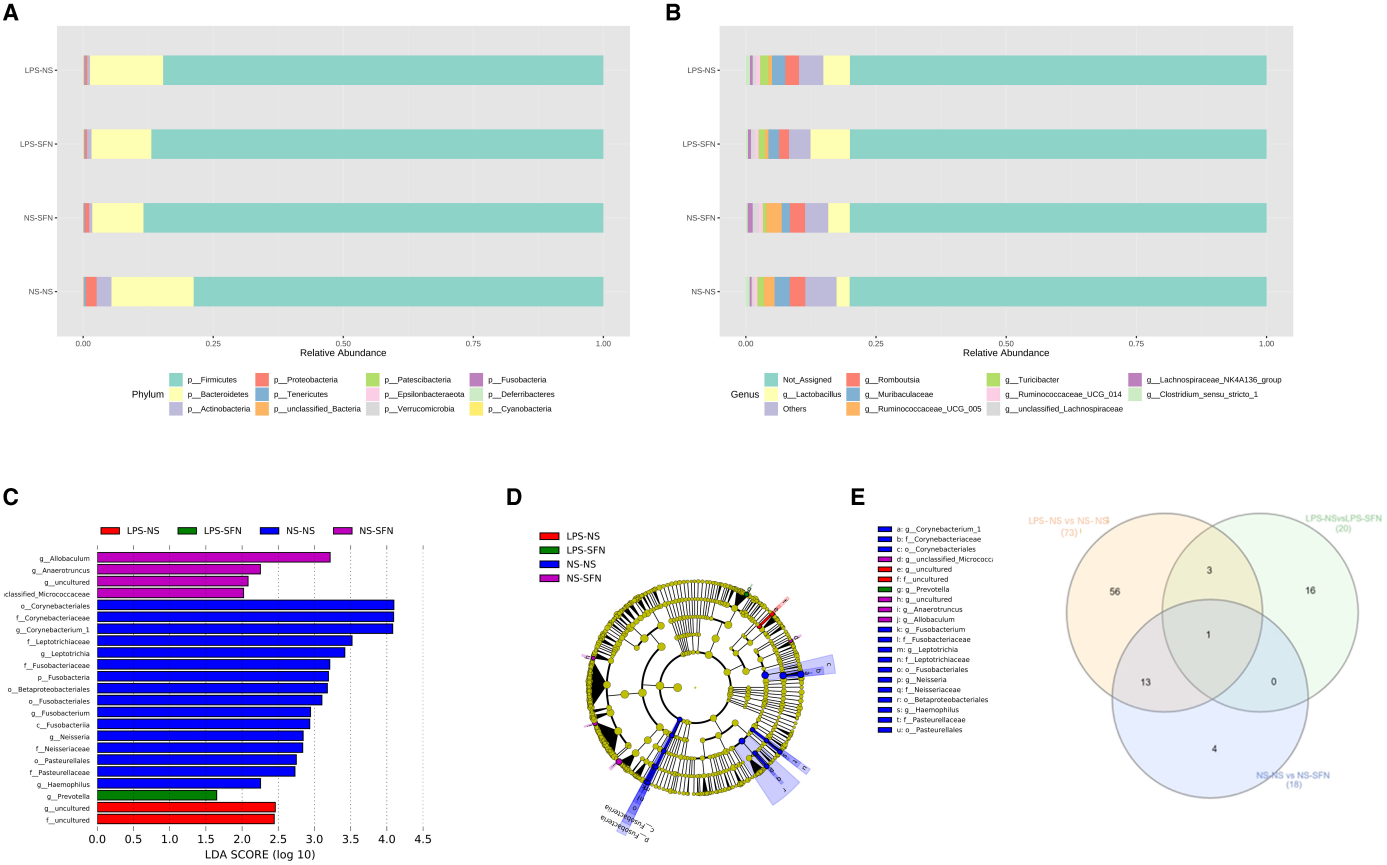
Relative abundance of differential taxa ASD-like rats and after SFN treatment. **(A)** Bar plots of the phylum taxonomic levels and genus taxonomic levels **(B)** among four groups (only the top 10 phylum bacteria are shown). **(C)** Histogram and cladogram **(D)** of the linear discriminant analysis (LDA) scores revealed distinct taxa in the gut microbiota among four groups. **(E)** Venn diagram showing the distribution of the OTUs among intergroup comparison.

We next investigated the dynamic changes of microbial composition in groups. At the phylum level, all groups showed a similar taxonomic composition, i.e., dominated by the phylum Firmicutes and Bacteroidetes, while the LPS-NS group had an increase in the mean Firmicutes/Bacteroidetes ratio (6.01 vs. 4.98) and a decrease in the abundance of Bacteroidetes compared to the NS-NS group. In the LPS-NS group, the abundance of Actinobacteria declined and SFN treatment ameliorated the reduction of Bacteroidetes and Actinobacteria (Figure 3A). Taxonomic compositions at the genus level were analyzed in the four groups (Figure 3B). The relative abundance of genus *Ruminococcaceae_UCG_005* enriched in the LPS-NS rats compared to the NS-NS rats. The relative abundance of genus *Lactobacillus* enriched in the LPS-SFN group (Figure 3B).

The LEfSe analysis, a method for identifying bacterial taxa as biomarkers using LDA with effect size measurements, was used to explore the significant differences at distinct microbial levels. The Fusobacteria (from the phylum to the genus *Fusobacterium*), Corynebacteriales (from the class to the genus *Corynebacterium 1*), Leptotrichiaceae (the family and the genus *Leptotrichia*), Neisseriaceae (the family and the genus *Neisseria*), Pasteurellales (the order and the family *Pasteurellaceae*), order Betaproteobacteriales, and genus *Haemophilus* enriched in NS-NS rats. Genus *Allobaculum, Anaerotruncus*, uncultured of Family_XIII, and unclassified_Micrococcaceaewere enriched in the NS-SFN group. Family uncultured and genus uncultured of order Coriobacteriales enriched in the LPS-NS group. Genus *Prevotella* displayed a relative enrichment in the LPS-SFN group (*p* < 0.05, Figures 3C, D).

To obtain deeper insight into microbiota alterations upon LPS or SFN administration, we analyze specific taxa in four groups by Venn diagram. As was shown in Figure 3E, three taxa were common in “LPS-NS vs. NS-NS” (Figure S1) and “LPS-NS vs. LPS-SFN” (Figure S1): phylum Fusobacteria, order Fusobacteriales, and class Fusobacteriia. Moreover, genus *Anaerotruncus* was common in “LPS-NS vs. NS-NS,” “LPS-NS vs. LPS-SFN,” and “NS-NS vs. NS-SFN” (Figure S1). These data suggested that SFN may exert a therapeutic effect by altering the abundance of some specific taxa.

### 3.4 Network analysis shows associations between GM and rats' social behavior

**Figure 4:**
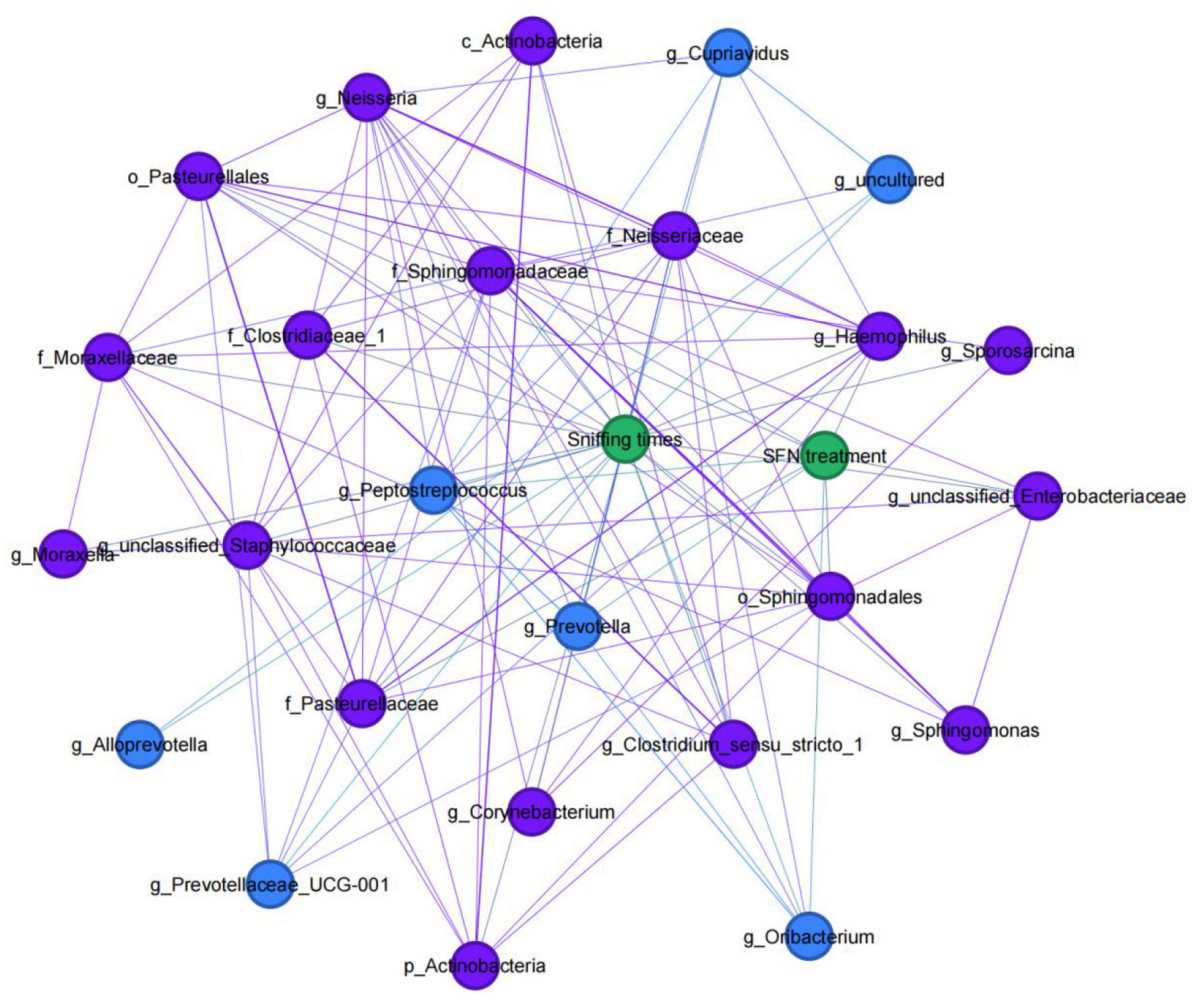
Social behavior-microbial network in MIA-induced ASD-like rat model. An undirected network from the microbiota to social behavior or SFN treatment of MIA-induced ASD-like rat model was built. Blue nodes indicate gut microbiota taxa that were positively correlated with sniffing times, and purple nodes indicate taxa that were negatively correlated with sniffing times.

Figure 4 shows the network analysis in the MIA-induced ASD-like rat model, and it showed associations between GM and rats' social behavior or SFN treatment. At the genus level, the relative abundance of *Alloprevotella* (*r* = 0.335, *p* = 0.04), *Prevotella* (*r* = 0.333, *p* = 0.041), *Prevotellaceae_UCG-001* (*r* = 0.387, *p* = 0.016), *Peptostreptococcus* (*r* = 0.346, *p* = 0.033), *Cupriavidus* (*r* = 0.421, *p* = 0.009), *uncultured* (*r* = 0.422, *p* = 0.08), and *Oribacterium* (*r* = 0.333, *p* = 0.041) was positively correlated with sniffing times, while the relative abundance of *Corynebacterium* (*r* = −0.359, *p* = 0.027), *Sporosarcina* (*r* = −0.322, *p* = 0.049), *unclassified_Staphylococcaceae* (*r* = −0.334, *p* = 0.04), *Clostridium_sensu_stricto_1* (*r* = −0.375, *p* = 0.02), *Neisseria* (*r* = −0.379, *p* = 0.019), *unclassified_Enterobacteriaceae* (*r* = −0.435, *p* = 0.006), *Haemophilus* (*r* = −0.421, *p* = 0.009), *Moraxella* (*r* = −0.333, *p* = 0.041), and *Sphingomonas* (*r* = −0.415, *p* = 0.001) was negatively correlated with sniffing times. In addition, of the taxa which had a correlation with sniffing times, we found that the genus *Prevotella* (*r* = 0.468, *p* = 0.003), genus *Peptostreptococcus* (*r* = 0.366, *p* = 0.024), and genus *Oribacterium* (*r* = 0.431, *p* = 0.007) were positively correlated with SFN treatment, while order Pasteurellales (*r* = −0.452, *p* = 0.004), family Sphingomonadaceae (*r* = −0.388, *p* = 0.016), family Pasteurellaceae (*r* = −0.452, *p* = 0.004), *genus unclassified_Enterobacteriaceae* (*r* = −0.342, *p* = 0.035), and *genus Haemophilus* (*r* = −0.501, *p* = 0.001) were negatively correlated with SFN treatment. In addition, of the taxa associated with SFN treatment, we found order Pasteurellales, family Pasteurellaceae, and *genus Haemophilus* enriched in the NS-NS group and *genus Prevotella* enriched in the LPS-SFN group.

### 3.5 Beneficial effect of SFN treatment in ASD patients

**Figure 5:**
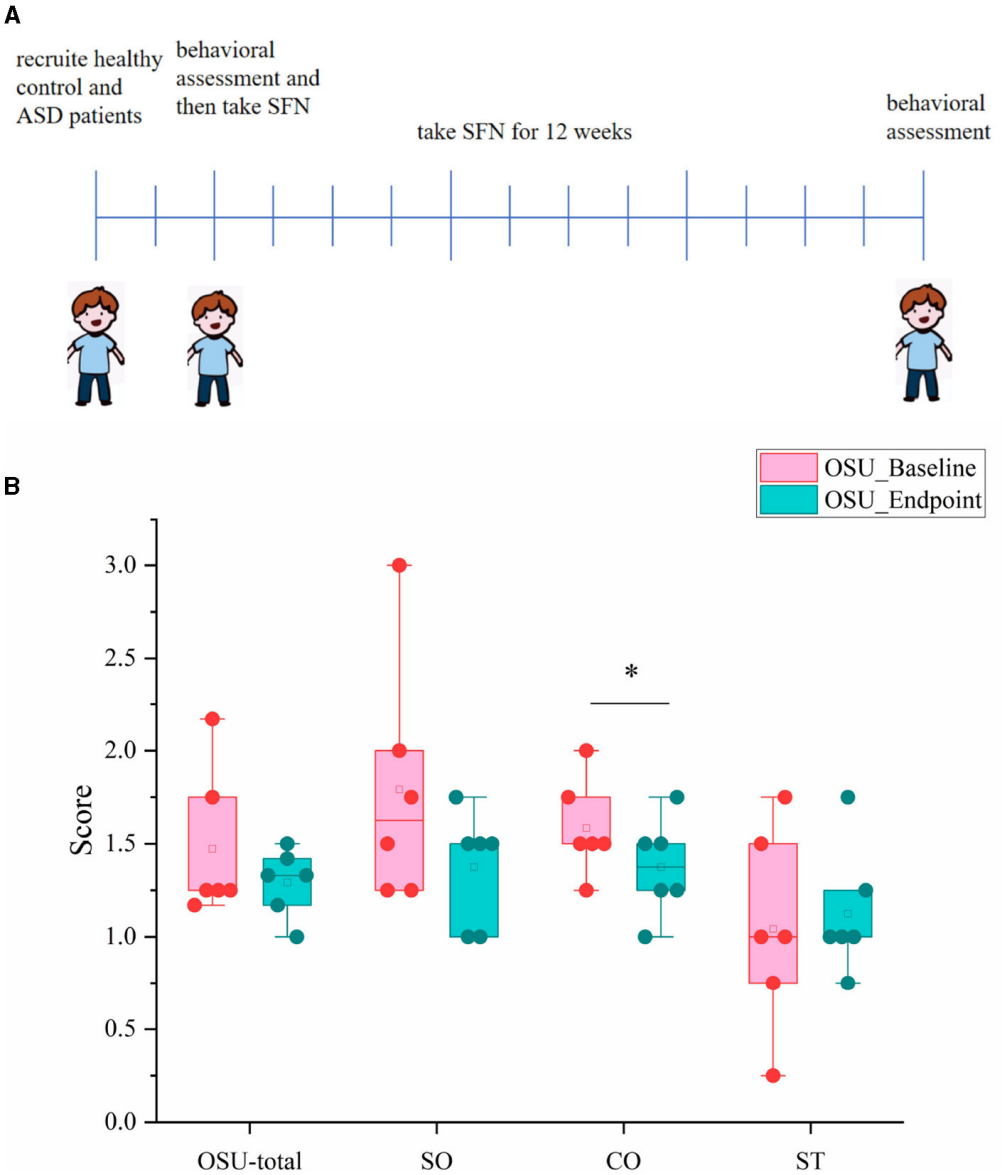
OSU scores and subscores of ASD patients at baseline and endpoint. **(A)** Schematic of experimental protocols. **(B)** OSU-total for total OSU behavioral scores, OSU-SO for social interactive OSU behavioral subscores, OSU-CO for non-verbal communicative OSU behavioral subscores, and OSU-ST for repetitive or ritualistic OSU behavioral subscores (*p < 0.05, paired Students' *t*-test).

We also conducted a 12-week clinical study to explore whether the SFN altered gut microbiome in ASD patients and ameliorated ASD by alteration of gut microbiota. The mean behavioral subscores and total scores and their changes of OSU of the six SFN-treated recipients from enrollment to the 12-week end of treatment are shown in Figure 5. Half of the participants experiencing improvement to SFN and the average OSU-CO scores of the SFN group had changed significantly at the 12-week endpoint (Figure 5B).

### 3.6 Gut microbial diversity altered in ASD patients

**Figure 6:**
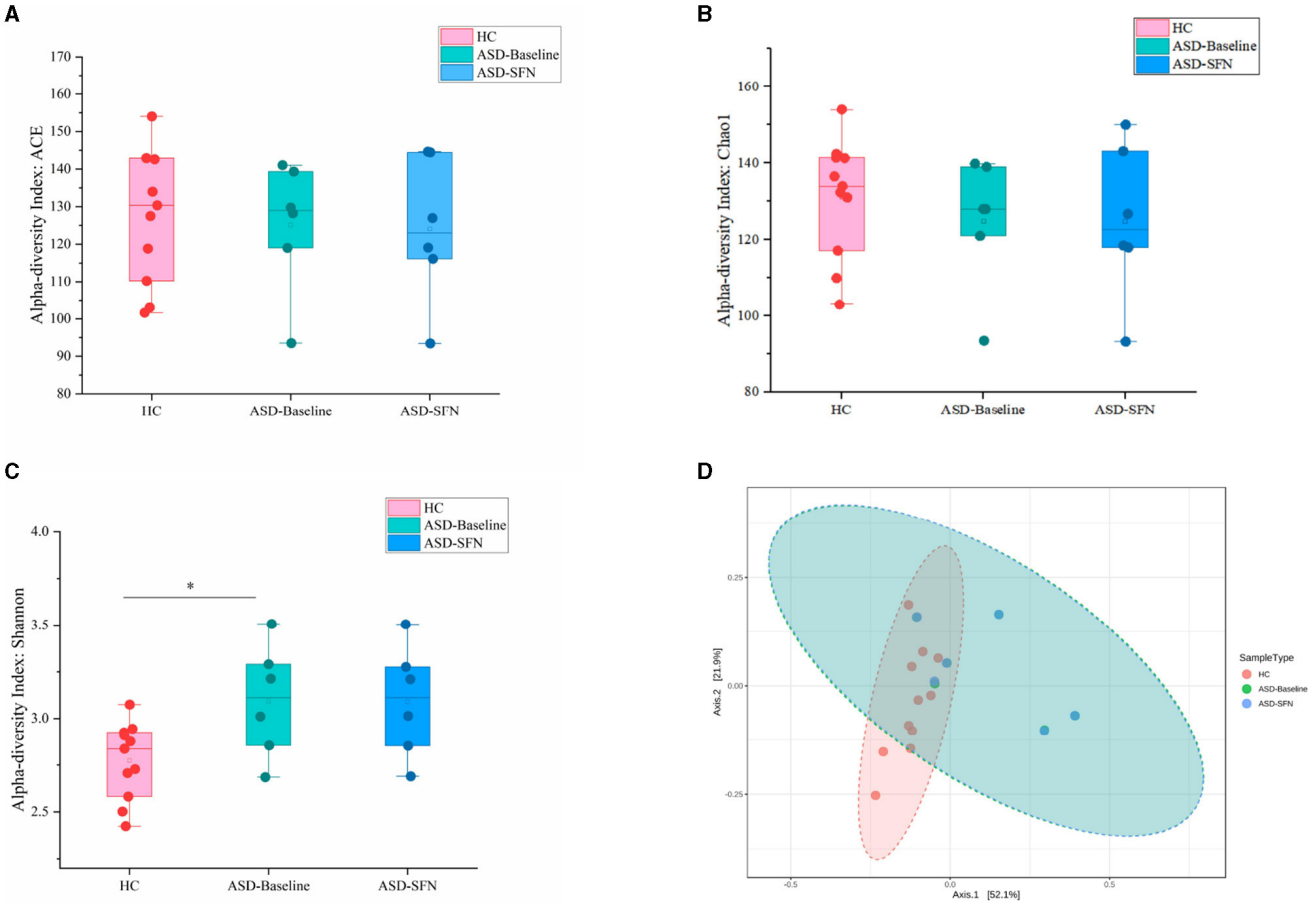
Diversity of the gut community altered in ASD patients. The ACE **(A)**, Chao1 **(B)**, and Shannon **(C)** used the Student's *t*-test and principal coordinates analysis (PCoA) of the samples with weighted UniFrac **(D)** (*p < 0.05).

The methods for assessing alpha and beta diversity were consistent with the animal part of this study. Figure 6 shows the alpha and beta diversity in ASD patients. In this study, gut microbial diversity estimated by the Shannon index, was greater in health controls (HCs) compared to untreated ASD patients (*p* = 0.03, Figure 6C). Beta diversity of ASD patients and HCs estimated by PCoA with weighted UniFrac showed that axis 1 accounted for 48.3% of the variation and axis 2 explained 25% of the variation. The fecal microbiota in untreated ASD and HCs could cluster separately (*p* = 0.002, Figure 6D). However, no diversity changes were associated with SFN treatment, which was estimated by paired Students' *t*-test between the ASD-Baseline group and the ASD-SFN group.

### 3.7 Changes of gut microbiota taxonomic composition in ASD patients

**Figure 7:**
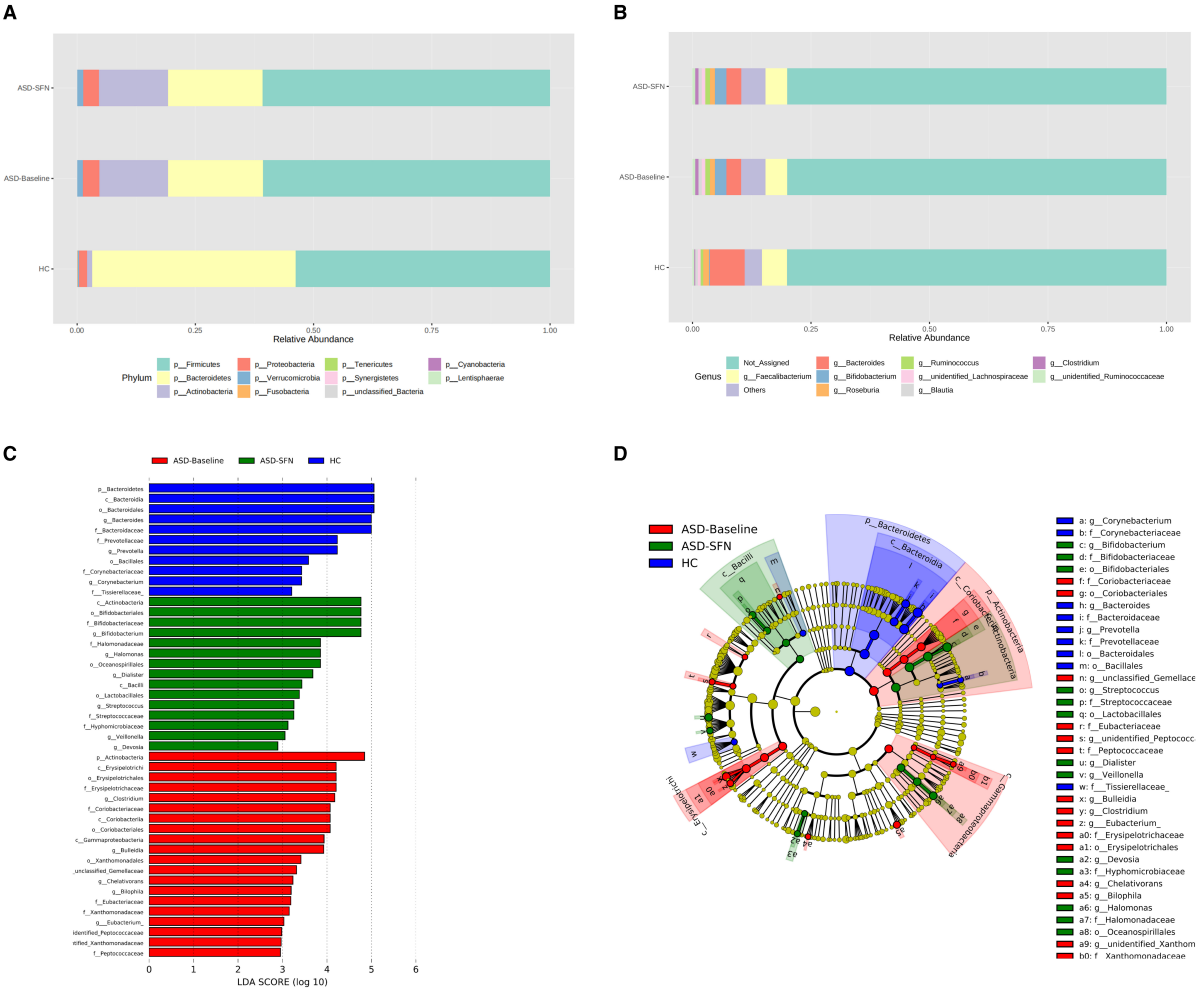
Differential taxa abundance in the gut microbiota. **(A)** Bar plots of the phylum taxonomic levels and genus taxonomic levels **(B)** among three groups (only the top 10 phylum bacteria are shown). **(C)** Histogram and cladogram **(D)** of the linear discriminant analysis (LDA) scores revealed distinct taxa in the gut microbiota among three groups.

The analysis of the gut microbiota composition at the phylum and genus levels showed specific differences in groups (Figures 7A, B). In terms of bacterial composition at the phylum level, untreated ASD patients had an increase in the mean Firmicutes/Bacteroidetes ratio (3.02 vs. 1.25) and a decrease in the abundance of Bacteroidetes compared to the HCs. The phylum *Bacteroidetes* was higher in HCs, while Actinobacteria enriched in ASD patients (Figure 7A). In terms of the genus level, all groups exhibited similar taxonomic communities, i.e., dominated by the genus *Faecalibacterium* and *Bacteroides*. The relative abundance of genus *Bacteroides* was significantly higher in the HCs compared to the ASD groups, while *Bifidobacterium* enriched in the ASD groups (Figure 7B).

The LEfSe analysis was used to explore the significant differences at distinct microbial levels among groups. We found that some gut microbiota had significant differences (Figures 7C, D). The Bacteroidetes (from the phylum to the genus *Bacteroides*), Prevotellaceae (the family and the genus *Prevotella*), Corynebacteriaceae (the family and the genus *Corynebacterium*), order Bacillales, and family Tissierellaceae enriched in HC group. The Erysipelotrichia (from the class to the family *Erysipelotrichaceae*), Coriobacteriia (from the class to the family *Coriobacteriaceae*), Xanthomonadales (from the order to the genus *unidentified Xanthomonadaceae*), Eubacteriaceae (the family and the genus *Eubacterium*), Peptococcaceae (the family and the genus *unidentified Peptococcaceae*), phylum Actinobacteria, class Gammaproteobacteria, and genus *Clostridium, Bulleidia, unclassified Gemellaceae, Chelativorans, and Bilophila* were found enriched in untreated patients. Bifidobacteriales (from the order to the genus *Bifidobacterium*), Halomonadaceae (the family and the genus *Halomonas*), Streptococcaceae (the family and the genus *Streptococcus*), class Actinobacteria and Bacilli, order Oceanospirillales and Lactobacillales*, and* genus *Dialister, Veillonella*, and *Devosia* enriched in the treated by SFN patients.

### 3.8 Network analysis of microbiome abundance alterations and improvements in ASD symptoms after SFN treatment

**Figure 8:**
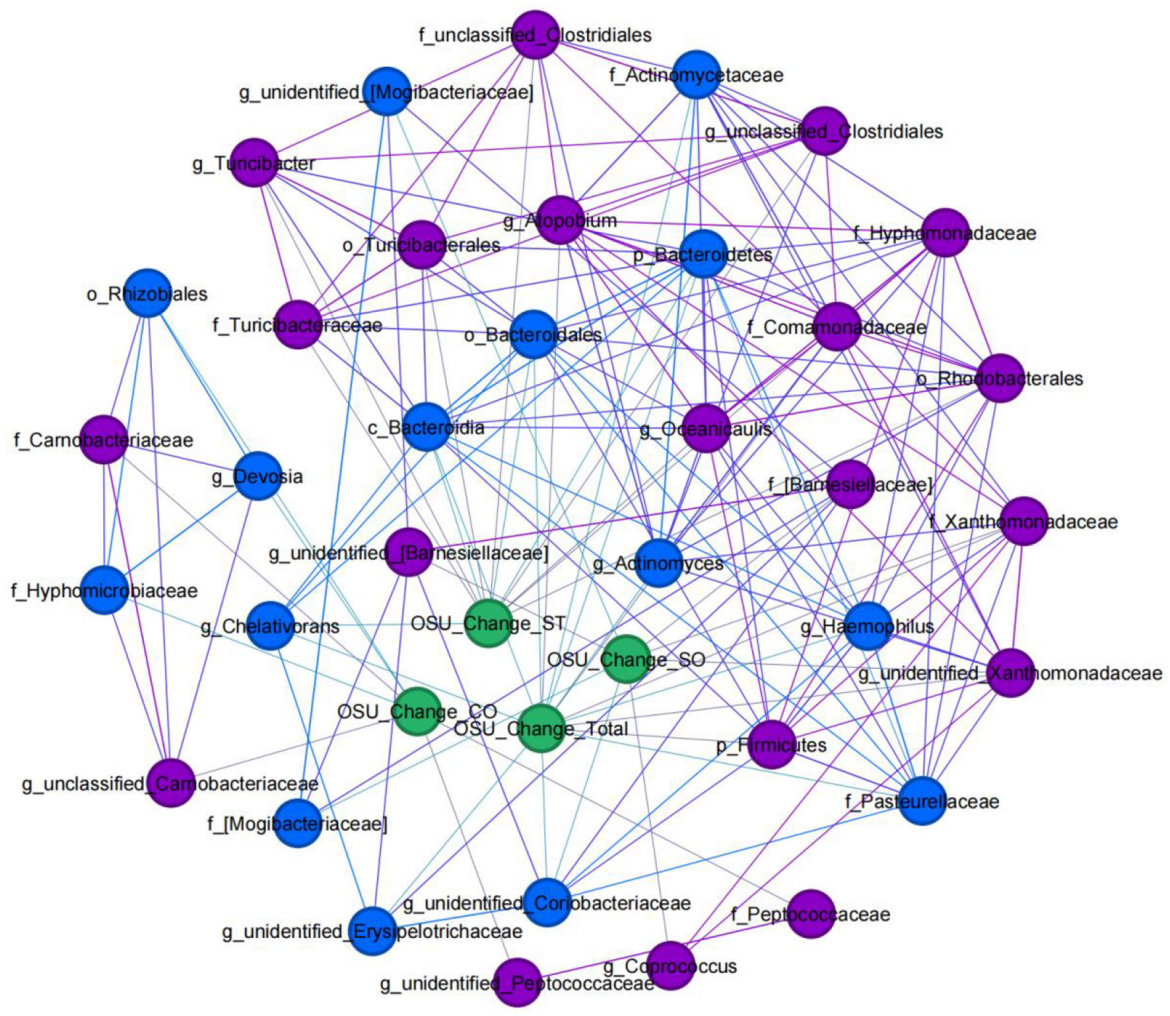
Clinical-microbial network in ASD patients with SFN treatment. An undirected network from microbiome abundance alterations to improvements in ASD symptoms after SFN treatment was built. Blue nodes indicate gut microbiota taxa that were positively correlated with OSU change, and purple nodes indicate taxa that were negatively correlated with OSU change. Alteration of the relative abundance of gut microbes: gut microbes' relative abundance at the endpoint was subtracted from the same gut microbes' relative abundance at time 0 (“Baseline”). OSU change: individuals' scores at endpoint were subtracted from the same individual's scores at time 0 (“Baseline”).

To elucidate the actual relationship between the alteration of the relative abundance of bacterial taxa on each taxonomical level and improvements in autistic symptoms after SFN treatment, we conducted a co-expression network analysis in ASD patients (Figure 8). At the genus level, the alteration of the relative abundance of *Atopobium* (*r* = 0.845, *p* = 0.034) and *unidentified_Xanthomonadaceae* (*r* = 0.812, *p* = 0.05) had a positive correlation with OSU_Change_Total, while *Actinomyces* (*r* = −0.845, *p* = 0.034), *unidentified_Coriobacteriaceae* (*r* = −0.928, *p* = 0.008), *unidentified_Erysipelotrichaceae* (*r* = −0.899, *p* = 0.015), *Chelativorans* (*r* = −0.845, *p* = 0.034), and *Haemophilus* (*r* = −0.886, *p* = 0.019) had a negative correlation with OSU_Change_Total. *unidentified_[Barnesiellaceae*] (*r* = 0.857, *p* = 0.029) *Coprococcus* (*r* = 0.928, *p* = 0.008) and *unidentified_Xanthomonadaceae* (*r* = 0.897, *p* = 0.015) were positively correlated with OSU_Change_SO, while *unidentified_Coriobacteriaceae* (*r* = −0.824, *p* = 0.044) and *unidentified_[Mogibacteriaceae]* (*r* = −0.868, *p* = 0.025) were negatively correlated with OSU_Change_SO. For OSU_Change_CO, *unclassified_Carnobacteriaceae* (*r* = 0.876, *p* = 0.022), and *unidentified_Peptococcaceae* (*r* = 0.822, *p* = 0.045) had a positive correlation with it, while *Devosia* (*r* = −0.939, *p* = 0.005) had a negative correlation with it. In addition, *Turicibacter* (*r* = 0.971, *p* = 0.001), *Oceanicaulis* (*r* = 0.857, *p* = 0.029), and *unclassified_Clostridiales* (*r* = 0.841, *p* = 0.036) were positively correlated with OSU_Change_ST, while *Chelativorans* (*r* = −0.823, *p* = 0.044) was negatively correlated with OSU_Change_ST.

Moreover, of the taxa which had co-association with ASD symptoms, we found phylum Bacteroidetes, class Bacteroidia, and order Bacteroidales were enriched in HC, family Peptococcaceae, family Xanthomonadaceae, genus *Chelativorans*, genus *unidentified_Peptococcaceae*, and genus *unidentified_Xanthomonadaceae* enriched in the ASD-Baseline group, and family Hyphomicrobiaceae and genus *Devosia* enriched in the ASD-SFN group. In addition, the family Pasteurellaceae and *the* genus *Haemophilus* were found in the network analysis in the rat model which was also associated with SFN treatment.

### 3.9 Commonly altered microbial taxa in both ASD-like rats and ASD patients

**Figure 9:**
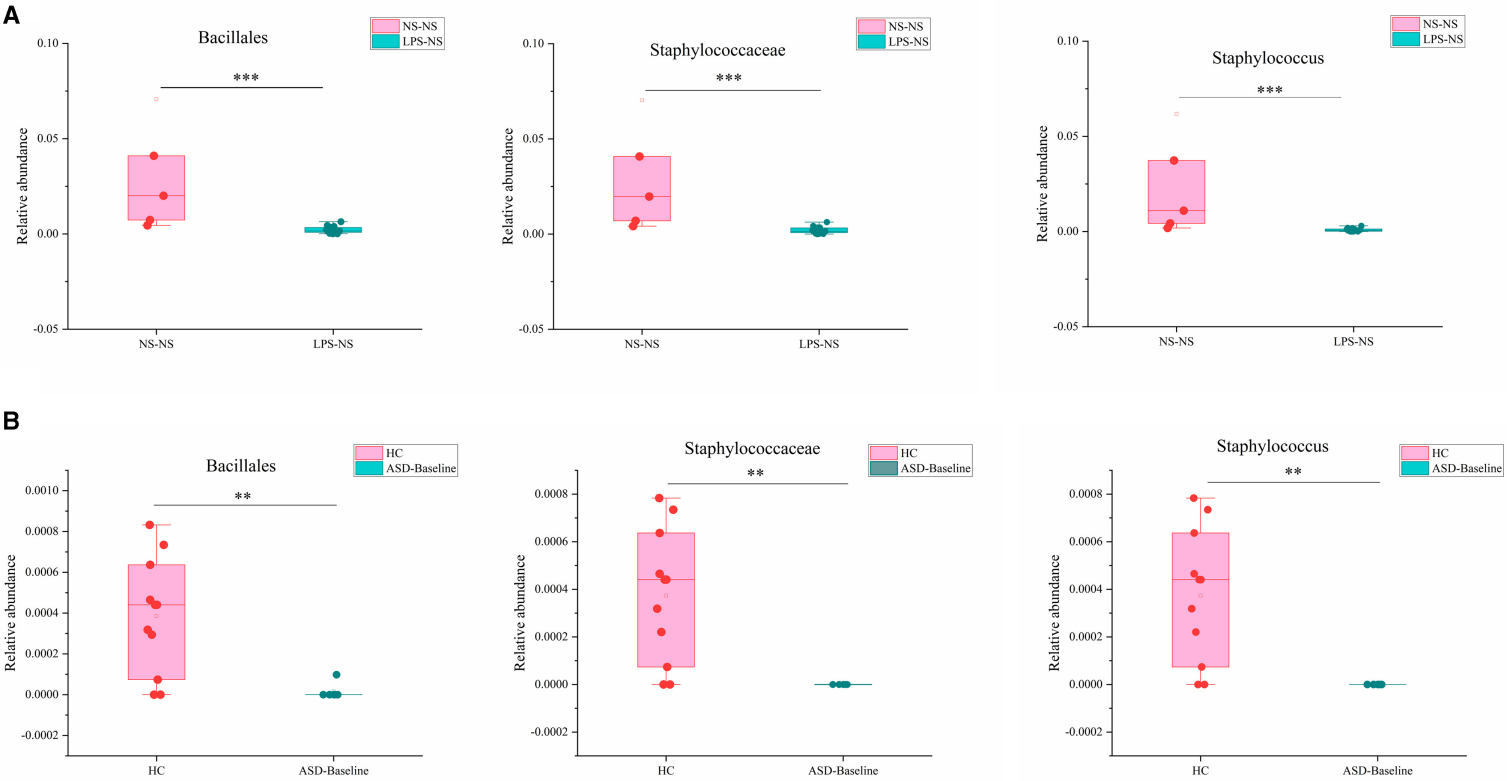
Overlapping altered microbial taxa in ASD-like rat models and ASD patient samples. **(A)** The overlap gut microbiota showed different relative abundances between the NS-NS group and the LPS-NS group. **(B)** The overlap gut microbiota showed different relative abundance between the HC group and the ASD-Baseline group (**p < 0.01 and ***p < 0.001, unpaired Students' *t*-test).

We next compared the gut microbiota which had significant differences between ASD-like rats and ASD patients and found three taxa had consistent changes in both “NS-NS vs. LPS-NS” (Figure S1) and “HC vs. ASD-Baseline” (Figure S2). The relative abundance of order Bacillales, family Staphylococcaceae, and genus *Staphylococcus* (*p* < 0.0001, 0.0002, and 0.0002, respectively, for the rats; *p* = 0.0076, 0.004, and 0.004, respectively, for the human) was lower in ASD-like rats and ASD patients (Figure 9). In our study, differences of the relative abundance of these several taxa were overlapped in different species, i.e., rat and human, but no overlap was found between species for SFN treatment-related differential microbiota.

## 4 Discussion

In the present study, we demonstrated that SFN treatment rescued the social deficits of ASD-like rats and ASD children. Additionally, SFN treatment-induced improvement in social deficits was associated with the relative abundance of gut microbial genera that differed significantly between groups in ASD-like rats. In addition, microbiome abundance alterations of gut microbes were associated with improvements in ASD symptoms of children with ASD after SFN treatment. Our study identified differences in the composition of the gut microbiota of ASD-like rats and ASD patients, which may contribute to autism-related behaviors, and these taxa are associated with improvements in symptoms of autism. These results suggested that the therapeutic effect of SFN may be related to gut microbiota.

We found that SFN treatment rescued the social deficits of MIA-induced ASD-like rats in the three-chambered test. Although current clinical studies suggest a role for SFN in the treatment of ASD, fewer studies have used animal models to explore its specific mechanisms, and only one study that used mice models of autism found the therapeutic effect of SFN (35). Using a MIA-induced ASD-like rat model and three-chamber social tests, we found the improvement effect of SFN on social behavior in ASD, and it may provide new ideas for future experimental animal studies. Furthermore, we explored the therapeutic effects of SFN on autism-related behaviors in children aged 4–7 years with ASD. Given our findings in animal models, we conducted a study with a small clinical sample with the aim of exploring the therapeutic effects of SFN in patients with ASD, focusing on the gut microbiota. Half of the participants had ASD symptom improvement after SFN administration, and there were significant changes in OSU-CO scores at the endpoint of 12 weeks in the SFN group. However, this is only a small sample of our attempts, and larger sample sizes will be needed in future to validate the therapeutic effects of SFN on ASD.

We found that the GM diversity of ASD-like rats and ASD patients was significantly different from that of the control group, which is consistent with previous studies (21, 22, 36–38), but we did not find that SFN had a significant effect on the GM diversity of the animals or clinical subjects. In addition, we found order Bacillales, family Staphylococcaceae, and genus *Staphylococcus* showed consistent alteration in both “NS-NS vs. LPS-NS” from the animal model and “HC vs. ASD-Baseline” from clinical samples. Their relative abundance was significantly lower in ASD-like rats and ASD patients and the alteration preserved cross-species, suggesting that these specific taxa may play an important role in the development of ASD and could potentially be biomarkers for identifying ASD. One previous study found a significantly higher abundance level of the genus *Staphylococcus* in the valproic acid rat model of the autism group than the control group, but this increase was observed only in females (39). It was different from our study, and this difference may be caused by gender. However, we did not find an overlap between taxa for SFN treatment-related differential microbiota in animal and human studies. This may be due to differences in the duration of SFN treatment (4 vs. 12 weeks), SFN administration patterns (intraperitoneal injection vs. oral), and the species itself.

Moreover, there were also some differences in the composition of the gut microbial community among ASD-like rats and ASD children. In a rat model, genus *Anaerotruncus* was found enriched in the LPS-NS group compared to the NS-NS group and the LPS-SFN group, but our study in ASD patients did not show an increased relative abundance of *Anaerotruncus*. Interestingly, we found beneficial bacteria, *Bifidobacterium* and *Lactobacillales*, enriched in ASD children treated by SFN. *Bifidobacterium* was found an increased abundance in children treated by microbiota transfer therapy which improved their gastrointestinal and autistic symptoms (40). In addition, the supplementation of *Lactobacillus reuteri*, which belongs to Lactobacillales, could alleviate ASD-like behaviors (27).

Of note, SFN administration caused an increase in the relative abundance of genus *Prevotella* in LPS-SFN rats. Although SFN treatment resulted in a significant increase in the abundance of *Prevotella* in rats, this result was not found in clinical samples, and previous studies have demonstrated that *Prevotella* had a decreased relative abundance in patients with ASD (41–43), as well as in the ASD mice model (44, 45), which was also demonstrated in our clinical samples. In children with ASD, microbiota transfer therapy altered the gut ecosystem, increasing overall bacterial diversity and the abundance of *Bifidobacterium, Prevotella*, and *Desulfovibrio* and improving gastrointestinal and autism symptoms (40). In the network analysis, we found a strongly positive association between genus *Prevotella* and the sniffing times of the three-chamber test in rats, suggesting increased *Prevotella* could rescue social deficits. All of these findings suggest that *Prevotella* plays an important role in the development and treatment of ASD.

Of note, family Pasteurellaceae and genus *Haemophilus* were found in the network analysis in both MIA-induced ASD-like rat models and ASD patients. Moreover, Pasteurellaceae was negatively correlated with SFN treatment in a rat model and had a positive correlation with OSU_Change_Total in ASD patients, which were consistent in both ASD-like rat models and ASD patients. Previous studies have demonstrated that Pasteurellaceae and *Haemophilus* had a decreased relative abundance in patients with ASD (46, 47). However, we did not find Pasteurellaceae, and *Haemophilus* had significant differences between ASD patients and HCs. It suggested that they may be related to the therapeutic efficacy of SFN and that future studies need to give them more attention.

Previous studies have shown that SFN could alleviate hyperuricemia by decreasing the relative abundance of the genus *Parasutterella* and increasing the relative abundance of the family Lactobacillaceae (31). It is worth noting that in our study, it was also found that SFN may alleviate the symptoms of ASD by regulating the relative abundance of these gut microbiota. Research has shown that compared to HCs, the abundance of genus *Parasutterella* was lower in patients with ASD (48, 49). We found that in a rat model, *Parasutterella* was enriched in the NS-SFN group compared to the LPS-SFN group (Figure S1), suggesting that SFN has the potential to ameliorate ASD symptoms by decreasing the relative abundance of *parasutterella*. It has been reported that order Lactobacillales, family Lactobacillaceae, and genus *Lactobacillus* have a lower relative abundance in the ASD mice model. Moreover, the administration of Lactobacillus reuteri, which belongs to Lactobacillaceae, could alleviate the symptoms of ASD (44). Interestingly, our study found that order Lactobacillales was enriched in the patients of the ASD-SFN group.

Taken together, our study was the first to investigate SFN's ability to ameliorate core ASD symptoms through modulation of the gut microbiota, and our study demonstrated that SFN treatment attenuates social deficits in rats and young children with ASD and some gut microbiota associated with the improvements in symptoms of ASD. These results suggested that SFN supplementation is a viable strategy for improving specific core symptoms of ASD and its therapeutic effects may be related to gut microbiota. It should be mentioned that there are some limitations in this study: First, studies were conducted in male rats and boys only; therefore, female rats and girls need to be included in future studies to determine whether gut microbiota and behavioral changes respond to SFN treatment in a sex-dependent manner; second, the sample size of our clinical study is relatively small and needs to be further expanded in future; finally, this study only uncovered a potential link between gut flora and the therapeutic effects of SFN on ASD, and the relationship between the mechanism of SFN and gut microbiota has not been systematically validated, the exact mechanisms of which need to be explored in further studies.

## 5 Conclusion

These data demonstrate that SFN treatment alleviates social deficits in MIA-induced ASD-like rats and ASD patients, and the improvements might be associated with gut microbiota. These data indicated that SFN, as a potential intervention targeting the gut microbiota, could provide a novel avenue for preventing and treating ASD. However, the mechanisms involved in the association of these microbiota with the therapeutic effects of SFN need to be further explored.

## Data availability statement

The original contributions presented in the study are publicly available. This data can be found at: https://www.ncbi.nlm.nih.gov/sra. For rats 16S rRNA sequencing data—BioProject ID: PRJNA1018541. For human 16S rRNA sequencing data—BioProject ID: PRJNA1018910.

## Ethics statement

The studies involving humans were approved by Ethics Committee of Second Xiangya Hospital. The studies were conducted in accordance with the local legislation and institutional requirements. Written informed consent for participation in this study was provided by the participants' legal guardians/next of kin. The animal study was approved by the Animal Users Care and Use Committee of Central South University. The study was conducted in accordance with the local legislation and institutional requirements.

## Author contributions

JY: Data curation, Writing – original draft. LH: Data curation, Writing – original draft. SD: Writing – original draft. HZ: Writing – original draft. XC: Writing – original draft. JO: Writing – review & editing, Funding acquisition. XZ: Writing – review & editing, Funding acquisition.
