# Supplementary material for: Therapeutic efficacy of sulforaphane in autism spectrum disorders and its association with gut microbiota: animal model and human longitudinal studies
Source: Front Nutr. 2024 Jan 8;10:1294057. doi: 10.3389/fnut.2023.1294057 (PMC10800504; doi:10.3389/fnut.2023.1294057)
Supplement: Supplementary file 1 [file Presentation_1.pdf]

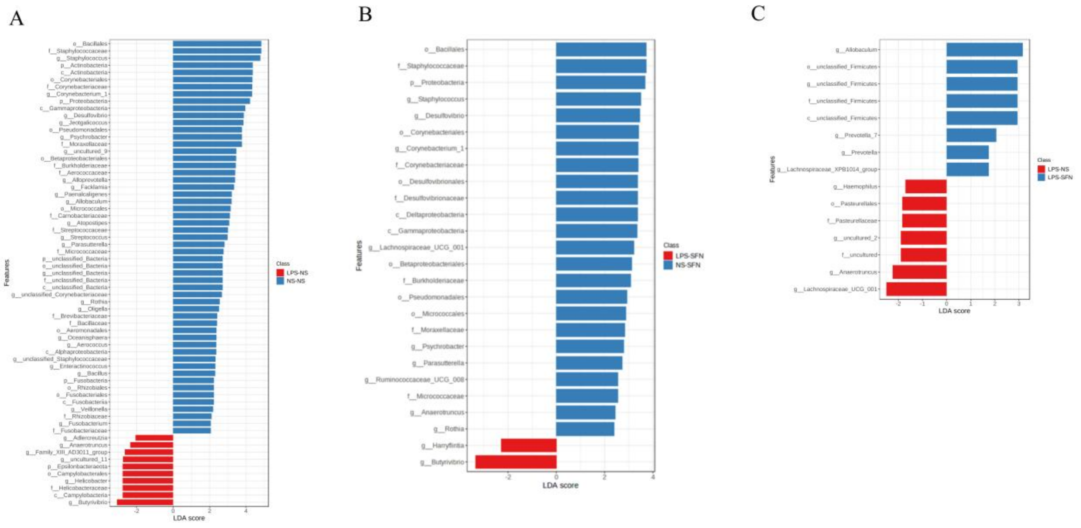

Figure S1 Taxonomic differences of fecal microbiota. Box plots of differentially abundant gut microbial taxa between LPS-NS group and NS-NS group (A), LPS-SFN group and NS-SFN group(B), LPS-NS group and LPS-SFN group(C).

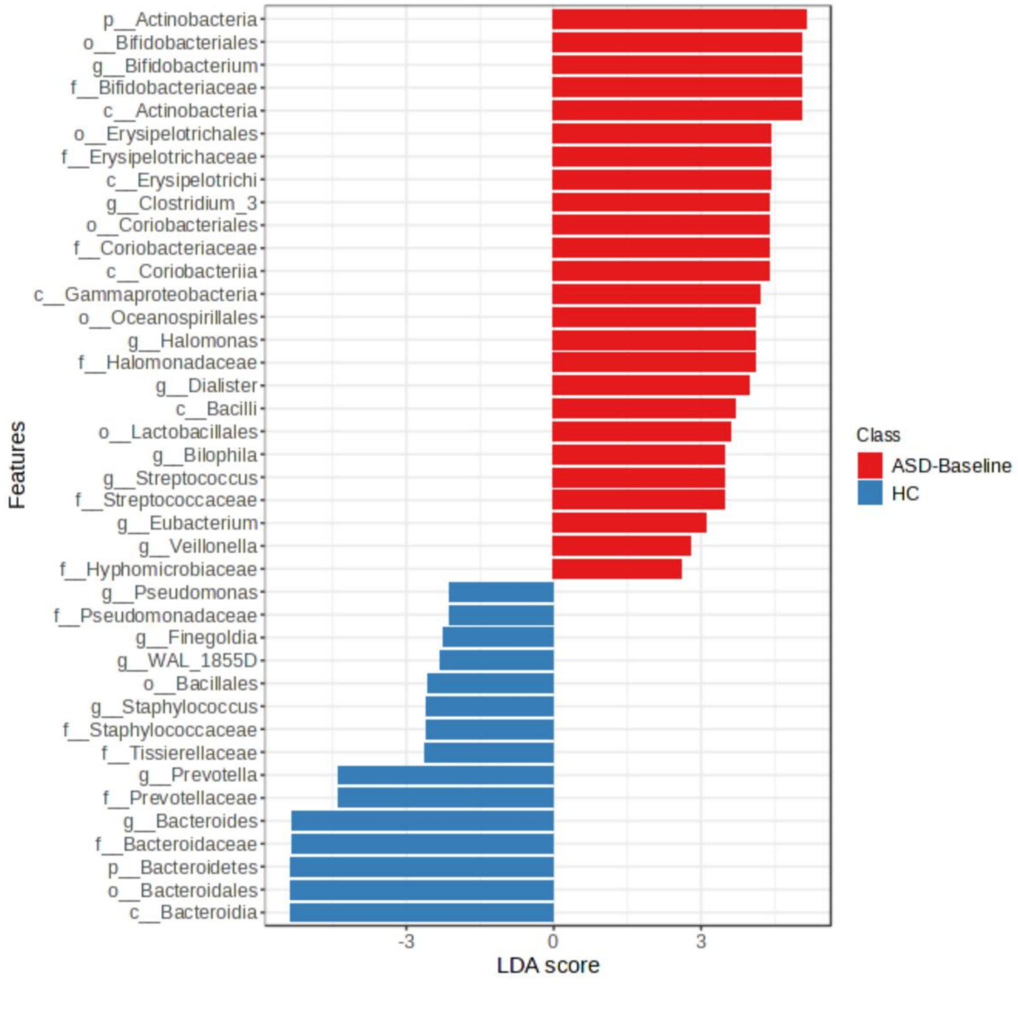

Figure S3 Box plots of differentially abundant gut microbial taxa between ASD-Baseline group and HC group.
